# Polarisation of Major Histocompatibility Complex II Host Genotype with Pathogenesis of European Brown Hare Syndrome Virus

**DOI:** 10.1371/journal.pone.0074360

**Published:** 2013-09-19

**Authors:** Christos Iacovakis, Zissis Mamuris, Katerina A. Moutou, Antonia Touloudi, Anne Sofie Hammer, George Valiakos, Themis Giannoulis, Costas Stamatis, Vassiliki Spyrou, Labrini V. Athanasiou, Maria Kantere, Tommy Asferg, Alexios Giannakopoulos, Charlotte M. Salomonsen, Dimitrios Bogdanos, Periklis Birtsas, Liljana Petrovska, Duncan Hannant, Charalambos Billinis

**Affiliations:** 1 Faculty of Veterinary Medicine, University of Thessaly, Karditsa, Greece; 2 Institute for Research & Technology-Thessaly, Larissa, Greece; 3 Department of Biochemistry & Biotechnology, University of Thessaly, Larissa, Greece; 4 Department of Veterinary Disease Biology, Faculty of Health and Medical Sciences University of Copenhagen, Copenhagen, Denmark; 5 Department of Animal Production, Technological Education Institute of Larissa, Larissa, Greece; 6 Institute for Bioscience, Aarhus University, Aarhus, Denmark; 7 Section for Fur Animal and Wildlife Diseases, National Veterinary Institute, Technical University of Denmark, Aarhus, Denmark; 8 Department of Medicine, University of Thessaly, Larissa, Greece; 9 Institute of Liver Studies, King’s College London, London, United Kingdom; 10 Department of Forestry and Natural Environment Administration, Technological Education Institute of Larissa, Karditsa, Greece; 11 Department of Bacteriology, Veterinary Laboratories Agency, Weybridge, United Kingdom; 12 School of Veterinary Medicine & Science, University of Nottingham, Nottingham, United Kingdom; University of Liverpool, United Kingdom

## Abstract

A study was conducted in order to determine the occurrence of European Brown Hare Syndrome virus (EBHSV) in Denmark and possible relation between disease pathogenesis and Major Histocompatibility Complex (MHC) host genotype. Liver samples were examined from 170 brown hares (hunted, found sick or dead), collected between 2004 and 2009. Macroscopical and histopathological findings consistent with EBHS were detected in 24 (14.1%) hares; 35 (20.6%) had liver lesions not typical of the syndrome, 50 (29.4%) had lesions in other tissues and 61 (35.9%) had no lesions. Sixty five (38.2%) of 170 samples were found to be EBHSV-positive (RT-PCR, VP60 gene). In order to investigate associations between viral pathogenesis and host genotype, variation within the exon 2 *DQA* gene of MHC was assessed. *DQA* exon 2 analysis revealed the occurrence of seven different alleles in Denmark. Consistent with other populations examined so far in Europe, observed heterozygosity of *DQA* (*H*_o_ = 0.1180) was lower than expected (*H*_e_ = 0.5835). The overall variation for both nucleotide and amino acid differences (2.9% and 14.9%, respectively) were lower in Denmark than those assessed in other European countries (8.3% and 16.9%, respectively). Within the peptide binding region codons the number of nonsynonymous substitutions (dN) was much higher than synonymous substitutions (dS), which would be expected for MHC alleles under balancing selection. Allele frequencies did not significantly differ between EBHSV-positive and -negative hares. However, allele Leeu-DQA*30 was detected in significantly higher (*P* = 0.000006) frequency among the positive hares found dead with severe histopathological lesions than among those found sick or apparently healthy. In contrast, the latter group was characterized by a higher frequency of the allele Leeu-DQA*14 as well as the proportion of heterozygous individuals (*P* = 0.000006 and *P* = 0.027). These data reveal a polarisation between EBHSV pathogenesis and MHC class II genotype within the European brown hare in Denmark.

## Introduction

The European brown hare (*Lepus europaeus*) is thought to have evolved on the open steppe grasslands of Eurasia and has adapted to mixed arable agriculture [1]. It is widespread throughout Europe and occurs in a variety of habitats ranging from Mediterranean to subarctic regions and from sea level to an altitude of c.2200 m. It has been successfully introduced into exotic temperate environments (e.g. Argentina, North America and New Zealand) and thrives on tropical and sub-Antarctic islands [2]. The European brown hare is the only *Lepus* species that exists in Denmark and although its numbers are declining, it remains one of the most important game species throughout the country [3].

In the 1980s outbreaks of a fatal disease associated with severe liver damage in hares occurred in Sweden [4], [5]. The etiological agent of this syndrome, named European brown hare syndrome (EBHS), was originally unknown but assumed to be either an infectious agent, most likely a virus, or a toxic chemical. In 1988, Lavazza and Vecchi identified, by electron microscopy, viral particles as the causative agent, which was classified as a calicivirus. Subsequently, EBHS has occurred in many European countries [6]–[23]. Nonetheless retrospective serological studies have demonstrated that the virus was present in Europe and other countries since as early as 1976 [4] while hunters in Scandinavia knew of the disease in the early 1970s [24]. The disease is currently endemic in Europe.

EBHS is a highly contagious and fatal disease that affects wild and farmed hares of the species *Lepus europaeus* and *Lepus timidus*. The adult animals, especially breeders, are more susceptible whereas animals under the age of 40 days remain unaffected. Morbidity may be as high as 100% and 35–80% mortality occurs 48–72 h after infection [25]. The disease is characterized by rapid progression, mild nervous symptoms, severe necrotic hepatitis, and circulatory dysfunction in various organs [4], [26]. The causative agent of EBHS is a small (30 to 35 nm) icosahedral, non-enveloped and hemagglutinating calicivirus [27]. Viral genome sequencing revealed that EBHS virus (EBHSV) shares a similar genomic organization to rabbit hemorrhagic disease virus (RHDV), but that the two viruses are distinct caliciviruses [28], [29]. EBHSV and RHDV, now classified in the new genus Lagovirus, recently created within the family Caliciviridae, have significant similarities in their epidemiology, clinical signs, and pathology [4].

The effectiveness of innate and adaptive immune responses, and hence the fitness of individuals, populations, and species, is driven by pathogen exposure history and the immunogenetic repertoire of Major Histocompatibility Complex (MHC) and non-MHC genes [30], [31]. MHC genes are the most polymorphic in vertebrates [32], some of which such as *DQA* and *DRB* within MHC II have hundreds of alleles described in human and in large vertebrate populations. Most variation in individual MHC genes is concentrated in regions coding for the extracellular domains that bind peptides derived from endogenous (MHC class I) or exogenous (MHC II) antigen processing [33]. In humans, some MHC alleles have been associated with resistance to specific diseases such as malaria, multiple sclerosis, hepatitis B and C, and papilloma virus [34], [35], [36]; Similar associations have been described for Marek’s disease in chickens [37], hanta virus in bank voles [38], respiratory diseases in sheep [39], Addison’s disease and demodectic mange in dogs [40], [41], and the severity of parasite burden in a variety of species including yellow-necked mouse [42], Malagasy mouse lemur [43], and three-spined stickleback [44].

Recently, an extensive study [45] on the level of MHC genetic diversity within and among natural populations of European brown hare from Greece, central Europe and Anatolia reported high polymorphism of the second exon of the *DQA* locus, one of the most polymorphic class II loci. In this study, specific alleles were identified that were unique to Greece or to the rest of Europe. In addition, the majority of the alleles were population specific, suggesting that gene flow was incapable of homogenizing the *DQA* gene pools completely and/or that differential selective pressures maintained these MHC allelic differences [45]. Infectious agents which show antigenic and genetic variation both over time, space and among populations have great potential for selection pressure on the evolution of MHC genes [46]. Newly emerged pathogens derived from antibody escape mutants, for example, may exhibit increased virulence by overcoming host immune responses and such pathogens may play a significant role in reducing the distribution and abundance of host populations over short timeframes (1–2 generations) through effects on survival and reproductive success [31].

The present study set out to determine the occurrence of EBHS and EBHSV in Denmark and to investigate possible associations between disease pathogenesis and MHC class II polarisation within the European brown hare. In order to do this, we examined the variations in the *DQA* gene encoding the alpha-1 domain which is integral to antigen processing. There is a substantial polymorphism for *DQA* genes in brown hares, but they express lower numbers of alleles than other MHC genes such as *DRB*. Although analysis of the MHC *DRB* gene has been performed we avoided any association analysis with EBHS because of two reasons (a) the excessive polymorphism detected with many alleles in low frequencies and (b) the existence of pseudogenes for the *DRB* (Koutsogiannouli et al. unpublished data, Smith et al 2011 [47]). Both parameters could blur the picture of susceptibility/resistance associations between *DRB* and EBHS. Hence, we proposed that DQA offered a greater potential for identifying complex associations between susceptibility or resistance to diseases related to MHC variation, as it suffers to a lesser extent from the analytical complications arising from high allelic diversity.

## Materials and Methods

### Ethics Statement

This study is part of European Union Seventh Framework Programme (2007–2013) large collaboration project under grant agreement no. 222633 (Novel Technologies for Surveillance of Emerging and Re-emerging Infections of Wildlife - WildTech). University of Nottingham is the Project Coordinator, and has received signed cooperation agreements with all the Project Partners, including National Veterinary Institute-Danish Technical University, for providing animal samples to the project. All samples used in this project represent archived material collected by Partners and other organisations for purposes other than this project as specified in deliverable D4.5/5.5 entitled “Guidelines for ethical sample collection” submitted to European Commission (26/02/2010, Dissemination Level: PP, Restricted to other programmeparticipants, including Commission Services).The majority of the Danish hares samples were collected for a biological project focused on population dynamics in cultured habitats from hunter-harvested animals. The project was co-funded by the Danish Nature Agency (Official Government Agency) and the hares samples were collected in accordance with European and Danish Hunting legislation (Lovbekendtgørelse nr. 930 af 24. September 2009 om Jagt og Vildtforvaltning). The remaining samples were from hares found dead and submitted for the Passive Danish Wildlife Disease surveillance program during the period 2005–2012. The specific organ samples were originally intended for *Francisella tularensis* screening and any remaining amounts of tissue were saved in the archive. Permission to use the samples in the current study was obtained in the course of the “WildTech” project, as stated above, by the National Veterinary Institute-Danish Technical University.

Since samples were obtained from tissue archives, there was no active capture, killing and sampling of wild animals specifically for this study. Research on animals as defined in the EU Ethics for Researchers document (European Commission, 2007, Ethics for Researchers - Facilitating Research Excellence in FP7, Luxembourg: Office for Official Publications of the European Communities, ISBN 978-92-79-05474-7) is not a part of the project.

### Samples Collection, RNA Extraction, PCR Amplification and Sequence Analysis of EBHS Virus

The liver samples tested in this study were obtained from 170 European brown hares, hunter-harvested or found sick or dead between 2004 and 2009, from various regions of Denmark, in the context of the Danish Wildlife Disease surveillance program. At necropsy, samples from liver were collected and, except for aliquots for RT-PCR, were fixed in neutral buffered 10% formalin, embedded in paraffin, sectioned at 4 to 5 µm and stained with haematoxylin and eosin. The samples were stored at the Technical University of Denmark, National Veterinary Institute, Section for Fur Animal and Wildlife diseases, and later submitted still frozen for virological examinations to the Laboratory of Microbiology and Parasitology, Faculty of Veterinary Medicine, University of Thessaly (Greece), where they were stored at −80°C.

After thawing, liver samples (25 mg of each) were homogenized and total RNA was isolated using the PureLink™ RNA Mini kit (Invitrogen Corporation, Carlsbad California, USA) according to the manufacturer’s instructions. RT-PCR was performed using reagents supplied with the SuperScript™ First-Strand Synthesis System for RT-PCR (Invitrogen Corporation, Carlsbad California, USA). Aliquots of 10 µl were initially formed by mixing 2 µl of RNA extraction product with 1 µl 10 mM dNTP mix, 5 µl Random hexamers 50 ng/µl and 2 µl DEPC-treated water. The RNA/primer mixture was incubated for 5 minutes at 65°C and then placed on ice for at least 1 minute. Subsequently in the above mixture added 10 µl of a premix to obtain a final concentration of 2 µl 10XRT buffer, 4 µl 25 mM MgCl_2_, 2 µl 0.1 M DTT, 1 µl 40 U/µl RNaseOUT™ and 1 µl 50 U/µl SuperScript™ II RT. Reverse transcription reactions were carried out at 42°C for 50 minutes., which were then terminated by heating for 15 min at 70°C. Finally added 1 µl 2 U/µl RNase H and incubated for 20 minutes at 37°C. The reactions stored at −20°C. Following RT, a PCR (20 µl) was performed by mixing 2 µl of the cDNA reaction with 2 µl 10X PCR Buffer, 2 µl 2,5 mM dNTP mix, 0,8 µl 50 mM MgCl_2_, 0,2 µl Taq DNA Polymerase (5 U/µl), 20 pmoles of each primer and up to 20 µl DEPC-treated water. Primers HEF 5′-CCGTCCAGCATTCGTCCTGTCAC-3′ (nt1 795–1817) and HEB 5′-CATCACCAGTCCTCCGCACCAC-3′ were selected from the VP60 gene of EBHSV (nucleotides 6423 to 6687 on the complete genome sequence of EBHSV GD, accession no. Z69620) [5]. After an initial denaturation period of 9 minutes at 94°C target DNA amplified in 35 cycles as follows: 45 sec at 94°C, 45 sec at 60°C and 1 min at 72°C. The mixtures were then brought to 72°C for 7 min. Ten µl of each RT-PCR product were analysed by electrophoresis on a 2% agarose gel and stained with ethidium bromide (0.5 µg/ml). A 100 bp DNA ladder was analyzed on the same gel to serve as a size marker. The expected sizes of the RT-PCR products were 265 bp.

In order to gain genetic information and evaluate phylogenetic relationships between the EBHSV isolates, PCR products from seven isolates sequenced in both senses with the forward and reverse PCR primers (described above) using the fluorescent BigDye Terminator Cycle sequencing kit v3.1 (Applied Biosystems), followed by fragment separation with a 3730×l DNA Analyzer (Applied Biosystems). Sequence analysis was performed on 220 nt (positions 6446–6665 on the complete genome sequence of EBHSV GD, accession no. Z69620) of a 265 bp RT-PCR fragment of the region coding for VP60. All the samples were analyzed twice and only high-quality sequences were used. Corresponding sequences from the seven isolates of the virus were aligned using the alignment algorithm of software package Sequence Navigator (Applied Biosystems). Nucleotide sequences from other 41 EBHSV isolates that were included in phylogenetic reconstructions were retrieved from the EMBL database. To determine the appropriate model of sequence evolution and statistically compare successively nested more parameter-rich models for this data set, the program MODELTEST Version 3.6 [48] was used. With a statistical significance of *P* = 0.01 the HKY85 model [49], with gamma correction, obtained the best likelihood score and was thus selected for the neighbour-joining (NJ) analysis. Parsimony and ML trees were constructed under the heuristic search option with 100 random-taxon-addition replicates and tree bisection–reconnection branch swapping, using PAUP* 4.0 [50]. A Bayesian analysis was also performed with MRBAYES version 3.1 [51], under the HKY85 model of sequence evolution. Depending on the data set, random starting trees run for 2×106 to 8×106 generations were used, sampled every 100 generations. Burn-in frequency was set to the first 25% of the sampled trees.

### Genetic Variation of Major Histocompatibility Complex

In order to investigate potential differentiation in genotype susceptibility to EBHSV, the 170 liver samples were also used for the detection of polymorphism of the (MHC) between the different individuals.

Total genomic DNA was extracted following the method previously described in Koutsogiannouli et al. 2009 [45]. Exon 2 locus of hare MHC DQA gene was amplified using the primers DQA-Fw:5′-GTTGGTGCCTATGGCATAAA-3′, DQA-Rv: 5′-AGCAGTAGAGTTGGAGCGTTT-3′ [52] with slight modifications. PCR reactions (30 µL) contained 200–500 ng of genomic DNA, 10× Taq buffer, 2 mM MgCl_2_, 0.2 mM of each dNTP, 25 pmoles of each primer and 1 U Taq polymerase (HyTest). The cycling conditions consisted of an initial denaturation at 95°C for 4 min followed by 35 rounds of denaturation at 95°C for 30 sec, annealing at 58°C for 40 sec and extension at 72°C for 40 sec, with a final extension at 72°C for 10 min using an Eppendorf thermal cycler.

All samples were screened for polymorphisms at exon 2 of DQA locus using single-strand conformation polymorphism analysis (SSCP) according to Koutsogiannouli et al. 2009 [45]. PCR products showing the same SSCP pattern were grouped and representative samples from each profile were purified with Qiagen purification kit. Homozygous samples were directly sequenced bi-directionally by Macrogen Inc. (S. Korea), while PCR products from heterozygous samples were ligated to pGEM T-Easy vector (Promega) and transformed into DH5a *E. coli* competent cells in order to separate the two alleles. After blue/white selection, seven positive clones were picked and grown in a small scale. Plasmid DNA was isolated (Eppendorf Fast Plasmid purification kit), subjected to PCR-SSCP and clones containing the correct different alleles were sequenced by Macrogen Inc. in either direction.

Nucleotide and amino acid sequences were aligned using ClustalW [53]. GENETIX software [54] was used to estimate the allelic frequency distribution, expected (*H*e) and observed (*H*o) heterozygosities, *F*st and genetic distances among populations. The relative rate of non-synonymous and synonymous substitutions was calculated according to Nei and Gojobori (1986), applying the correction of Jukes and Cantor for multiple hits using MEGA4 [55]. MEGA was also used to calculate pairwise and overall nucleotide and amino acid differences and to construct the UPGMA and the neighbour-joining tree using the genetic distances of Jukes and Cantor [56] with 10000 bootstrap replicates. Tests for association between frequency distributions and associations of each allele and genotype in EBHSV positive or negative for dead or not dead animals under study were performed using the Cochran–Armitage trend test. The groups for analysis were inspected for normality and pair-wise comparisons were performed with the non-parametric Mann–Whitney test. All the analyses were performed using SPSS 14 (Inc., Chicago, IL, USA). A *P*≤0.05 was used to identify a significant result.

Hybridization followed by introgression of mtDNA between *Lepus* species in Europe is a common phenomenon [57]–[63]. We therefore verified whether introgressed individuals occurred among brown hares from Denmark, using methods of mtDNA analysis previously described [64], [65].

## Results

### Pathological Findings

Necropsy and histopathological examinations, conducted at the Technical University of Denmark, National Veterinary Institute, Section for Fur Animal and Wildlife diseases, revealed pathological findings consistent with EBHS in 18 (27.7%) of the 65 positive hares (64 dead hares and one showing abnormal behavior). The pathology was characterized by pulmonary congestion, oedema and haemorrhage and tracheal pus was frequently observed. Bloody exudates around the nostrils, conjunctivitis and congestion of the oral cavity mucosa were noticed in some cases. The liver in most cases was swollen with megnut discoloration and decreased texture, with congestion and diffusely scattered petechial bleeding. The spleen was congested and enlarged with degreased texture. Congestion was also observed in the kidneys. The most prominent and consistent histopathological lesions were found in the liver. Severe acute hepatitis and necrosis with periportal infiltration of lymphocytes and macrophages were often seen. Additionally vacuolization and areas with lipid accumulation in hepatocytes were observed. The lungs were characterized from congestion and in some cases there were accumulation of white blood cells. In the spleen massive congestion was observed and lymphoid reaction in one case. Kidney congestion was seen as well. Regarding EBHSV positive hares without typical lesions of EBHS, 23 (35.4%) had liver lesions not typical of the syndrome, 13 (20%) had other lesions and the eleven (16.9%) apparently healthy hares, that were shot during hunting, had no lesions.

### Virological Findings

**Figure 1:**
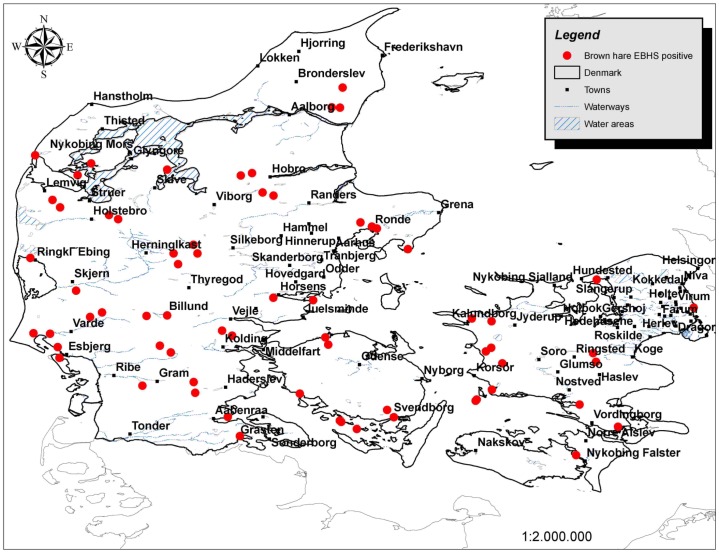
Location map of EBHSV positive *Lepus europaeus* individuals. Location map of the 65 *Lepus europaeus* individuals sampled throughout Denmark that were affected by the virus of the European Brown Hare Syndrome (EBHSV).

**Figure 2:**
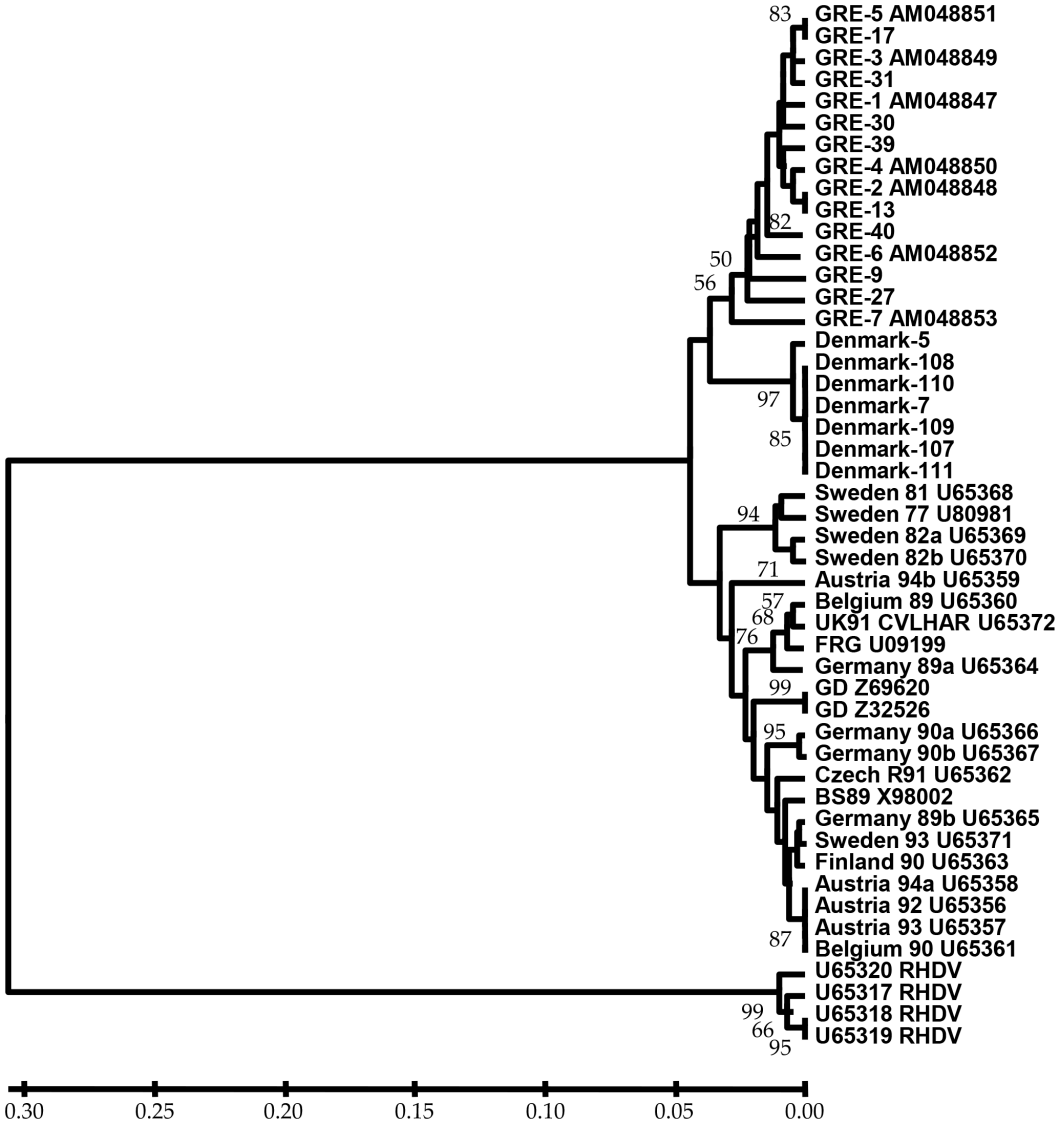
Phylogenetic tree of the EBHSV isolates. Phylogenetic tree resulting from the Bayesian analysis, clustering the seven Danish isolates identified in this study combined with sequences available from the EMBL database. The topology of the clusters was similar for the NJ tree. Numbers on branches at the internodes of the clusters correspond to posterior probabilities from the Bayesian analysis. At the end of the branches the designation and origin of the EBHS viruses studied. Probabilities below 50% are omitted.

Sixty five out of 170 brown hares were found positive for the EBHSV. Among them, 35 were found dead, 19 sick or with abnormal behavior and 11 apparently healthy hares were shot during hunting. EBHSV positive hares were distributed all over Denmark (Figure 1). Seven out of the 65 isolates from Denmark were phylogenetically analyzed together with 15 Greek isolates (seven previously described and eight new [23]), 22 other EBHSV and four RHDV isolates retrieved from the EMBL database. The 44 EBHSV and four RHDV isolates clearly separated as two distinct members of the Caliciviridae family, with an average genetic distance between the two groups of 0.606. Phylogenetic analysis showed that all isolates from Denmark formed a single genetic lineage, distinct from the Greek and other European isolates (Figure 2). The average genetic distances were 0.082 between Danish and the other European, 0.075 between Danish and Greek and 0.096 between Greek and European isolates.

### Variation in Exon 2 DQA Gene

Of the 170 examined brown hares, 158 yielded a PCR product (58 EBHSV positive and 100 EBHSV negative) (Table 1). Net PCR product, exclusive of the primers, corresponded to residues 8–76 of DQα chain according to the novel numbering system proposed by Bondinas et al. [45], [66]. No insertions/deletions or stop codons were detected indicating that all sequences identified could form functional molecules. Analysis of sequence variation of DQA exon 2 revealed the occurrence of seven different alleles among the 158 *L. europaeus* specimens. Of these alleles three were identical with alleles previously detected in Europe and reported in Koutsogiannouli et al. 2009 [45] namely Leeu-DQA*14, 30 and *42*. Alleles Leeu-DQA*14 and *30* are the most abundant, showing a wide distribution across Europe. Allele Leeu-DQA*42 has been detected so far in France, Netherlands and Great Britain. The remaining four alleles were unique in Denmark and referred to as Leeu-DQA*51 to Leeu-DQA*54 (GenBank accession numbers: JX261937–JX261940). In Denmark, allele Leeu-DQA*30 was highly abundant with a frequency of 0.608 followed by the alleles Leeu-DQA*14 (0.234), Leeu-DQA*52 (0.120), Leeu-DQA*53 (0.025), Leeu-DQA*42 (0.006), Leeu-DQA*51 (0.003) and Leeu-DQA*54 (0.003). The authenticity of rare alleles’ sequences was verified by repeated PCR and cloning. Alleles Leeu-DQA*30, *52* and *53* were present both in homozygous and heterozygous status. Alleles Leeu-DQA*14 and *42* were found only in homozygosity, with allele Leeu-DQA*42 to be present only in one hare. Finally, alleles Leeu-DQA*51 and *54* were found only in heterozygosity and in only one hare each. As for all populations examined so far in Europe for DQA variation [45] observed heterozygosity (*H*_o_ = 0.127) was clearly lower than expected heterozygosity (*H*_e_ = 0.560). This could have resulted from the presence of alleles that could not be amplified (null alleles), such that heterozygous individuals appeared as homozygotes. However, random PCR analysis of homozygous samples, using internal primers, resulted in the same profile (data not shown). Another possible explanation is that samples considered to be homozygous were heterozygous at sites outside of exon 2, resulting in underestimated level of heterozygosity.

**Table 1:** Groups of Danish brown hares individuals according to the presence of EBHSV and the variation in exon 2 DQA gene.

DQA genotype	EBHSV^−^		EBHSV^+^		EBHSV^+^			
					Dead		not Dead	
	N	%	N	%	N	%	N	%
Leeu-DQA*30	57	57	31	53.45	15	83.33	16	40
Leeu-DQA*14	22	22	15	25.86	2	11.11	13	32.5
Leeu-DQA*52	8	8	2	3.45	0	0.00	2	5
Leeu-DQA*53	0	0	1	1.72	0	0.00	1	2.5
Leeu-DQA*42	1	1	0	0.00	0	0.00	0	0
Leeu-DQA*30/Leeu-DQA*51	1	1	0	0.00	0	0.00	0	0
Leeu-DQA*30/Leeu-DQA*52	8	8	5	8.62	1	5.56	4	10
Leeu-DQA*30/Leeu-DQA*54	0	0	1	1.72	0	0.00	1	2.5
Leeu-DQA*52/Leeu-DQA*53	3	3	3	5.17	0	0.00	3	7.5
**Total**	100		58		18		40	

Numbers (N) and percentages of Danish brown hares assessed for the four groups defined in this study: EBHSV negative (EBHSV^−^), EBHSV positive (EBHSV^+^), susceptible (EBHSV^+^ Dead), resistant (EBHSV^+^ not Dead).

MtDNA analysis for introgression showed that 7% (12/170) of *L. europaeus* individuals were introgressed by the most common *L. timidus* mtDNA haplotype. This percentage fit well to earlier findings [62], [63], [67]. However, DQA genotype constitution did not differ between introgressed and non-introgressed hares, since introgressed individuals bore the most common alleles [Leeu-DQA*30 (0.611), Leeu-DQA*14 (0.222), Leeu-DQA*52 (0.110) and Leeu-DQA*53 (0.066)].

Thirty out of 212 (14.15%) nucleotide and 18 out of 70 (25.71%) amino acid positions were variable. The number of pairwise nucleotide differences between pairs of alleles ranged from 0.2% (Leeu-DQA*14 vs. Leeu-DQA*52) to 5.2% (Leeu-DQA*15 vs. Leeu-DQA*30) with an average of 2.9% and the number of amino acid differences from 0% (Leeu-DQA*14 vs. Leeu-DQA*52) to 26.0% (Leeu-DQA*15 vs. Leeu-DQA*30) with an average of 14.9%. Alleles Leeu-DQA*14 and Leeu-DQA*52 gave rise to identical amino acid sequences. Interestingly, the overall variations for both nucleotide and amino acid differences (2.9% and 14.9%, respectively) were clearly lower in Denmark than those assessed in other European countries (8.3% and 16.9%, respectively) [45].

**Figure 3:**
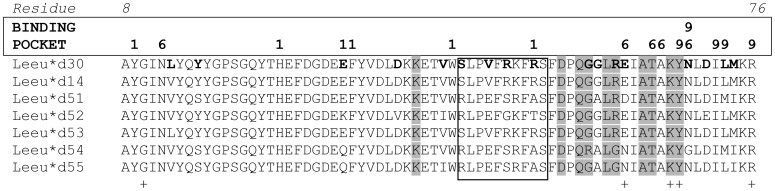
DQA alleles (residues 8–76) of *Lepus europaeus.* DQA alleles (residues 8–76) of *Lepus europaeus.* Polymorphic residues are in bold. Residues contributing to the formation of binding pockets P1, P6 and P9 are indicated. Shaded residues are putative TCR contacts. Crosses indicate residues with hydrogen bonds to the peptide. The boxed area determines αβ pairing.

Among the residues contributing to the formation of the peptide-binding region (PBR) pockets and binding of antigen via hydrogen bonds (PBR residues, [66]), five (33.33%) out of 15 amino acid positions were variable (Figure 3). Moreover, five out of 10 residues responsible for αβ chain pairing were polymorphic, along with three out of 10 residues between positions 39–68 that are involved in T-cell receptors (TCRs) contact.

**Figure 4:**
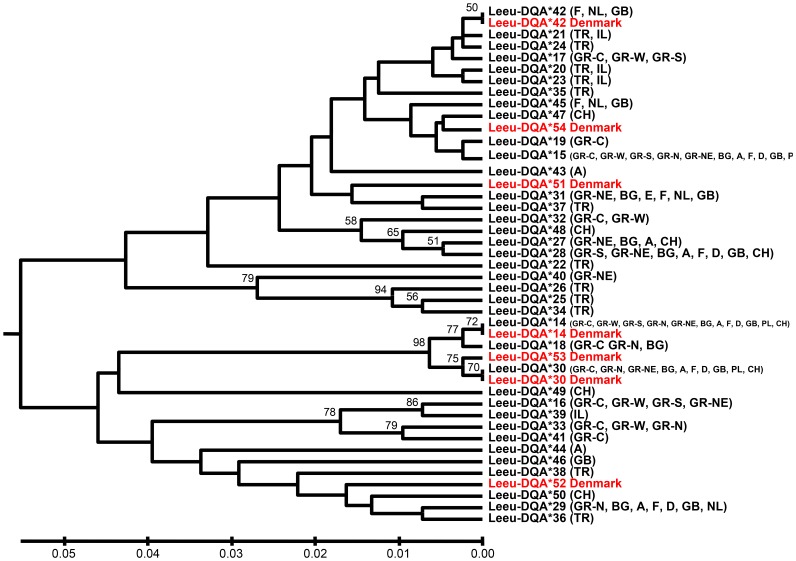
UPGMA phylogenetic tree of the eight *Lepus europaeus* DQA exon 2 alleles. UPGMA phylogenetic tree resulting from the analysis of the eight *Lepus europaeus* DQA exon 2 alleles identified in this study, together with sequences assessed in the study of Koutsogiannouli et al. 2009 available from GenBank. Numbers indicate the percentage bootstrap support (10000 replicates). The evolutionary distances were computed using the maximum composite likelihood method and are in the units of the number of base substitutions per site. The populations in which each allele was present are given abbreviated in parentheses. (The neighbour-joining tree produced similar results with comparable bootstrap values).

Figure 4 depicted the relationships among the seven alleles identified in the present study and the 37 DQA *L. europaeus* alleles that had been published previously [45] by the construction of a UPGMA tree, using the maximum composite likelihood method (a not shown neighbour-joining tree produced similar results with comparable bootstrap values). As expected, no separation of alleles on the basis of geographical distances was observed and the phylogenetic analysis showed that population-specific alleles did not cluster together. This sharing of alleles among distant populations are characteristic features of some MHC loci [68].

Within the PBR codons the number of nonsynonymous substitutions (dN) was much higher than that of synonymous substitutions (dS) (dN = 0.153±0.051 vs. dS = 0.062±0.036), which would be expected for MHC alleles under balancing selection. These differences favour a hypothesis of positive selection with a statistically significant *P*-value of 0.041 and strengthen the assumption that DQA loci in brown hare is functional and involved in antigen presentation, which is not always the case as seen in a recent study on MHC genes in bank voles [38]. However, for non-PBR codons, the ratio of synonymous vs. nonsynonymous substitutions was reversed (dN = 0.062±0.015 vs. dS = 0.088±0.027), and a trend towards purifying selection was detected as has been described previously by Hughes & Nei [69].

### Susceptibility to the EBHSV and Variation in Exon 2 DQA Gene

**Figure 5:**
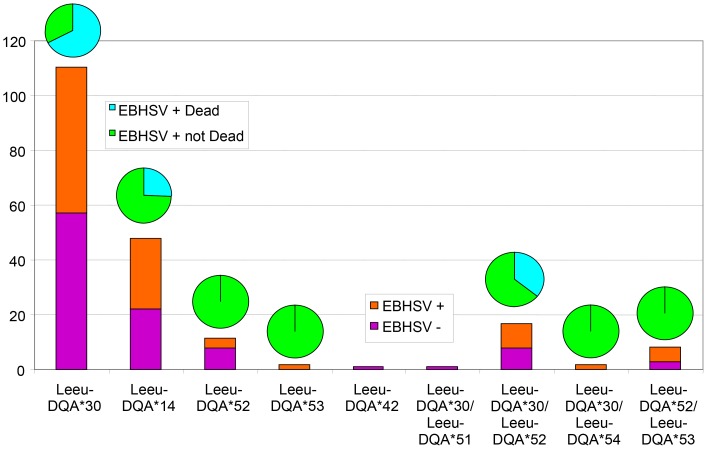
Percentages indicating the occurrence of the homogygous and heterozygous *DQA* genotypes. Percentages indicating the occurrence of the homogygous and heterozygous *DQA* genotypes in the four groups of *Lepus europaeus* sampled in Denmark. Bars indicate the occurrence of each genotype in non affected or affected animals. Pies above bars indicate the occurrence of each genotype among the affected *L. europeaus* individuals in susceptible animals found dead with lesions typical to the EBHS in various organs or resistant not dead animals.

For the purpose of the study, susceptibility to the EBHSV defined as the occurrence of deaths in association with the intensity of lesions typical to the EBHS in various organs. Therefore, the 158 individuals examined, both for the presence of EBHSV and the variation in exon 2 DQA gene, were assigned in four groups (Table 1). Distribution and frequencies of alleles as well as levels of heterozygosity were not significantly different between EBHSV positive and negative hares (*P* = 0.494 for the totality of the genotypes, *P* = 0.344 for homozygote genotypes and *P* = 0.404 for heterozygote genotypes). However, there was a significant difference of alleles’ distribution and frequencies within the group of EBHSV positive hares and between those found dead with severe histopathological lesions and those found sick or apparently healthy (*P* = 0.000006 for the totality of the genotypes, *P* = 0.000006 for homozygote genotypes and *P* = 0.027 for heterozygote genotypes) (Table 1 and Figure 5). Among the 18 positive hares that were found dead with severe histopathological lesions indicative of the syndrome, 15 were homozygous for the allele Leeu-DQA*30, two homozygous for the alleles Leeu-DQA*14 and *52* and one heterozygous for the alleles Leeu-DQA*30 and *52* (Table 1). The remaining 47 positive hares that were found sick or apparently healthy with lesions not typical to the syndrome or with no specific lesions, 16 were homozygous for the allele Leeu-DQA*30, 15 homozygous for the allele Leeu-DQA*14, five heterozygous for the alleles Leeu-DQA*30 and *52*, three heterozygous for the alleles Leeu-DQA*52 and *53,* two homozygous for the allele Leeu-DQA*52, one homozygous for the allele Leeu-DQA*53 and one heterozygous for the alleles Leeu-DQA*30 and *54* (Table 1). Even when the analysis was restricted to the two most common alleles Leeu-DQA*30 and 14 their distribution among the two groups of EBHSV positive hares showed significant variations (*P* = 0.000006). The frequency of the allele Leeu-DQA*30 was twofold higher within the EBHSV positive “susceptible” group and that of the allele Leeu-DQA*14 threefold higher in the EBHSV positive “resistant” group (Table 1, Fig. 5).

### Pocket Variants

The PCR product yielded in this study codes for fifteen of the sixteen residues at the α1 domain which contributes to the shaping of antigen pockets P1, P6 and P9 (PBR residues, [66]). The DQA sequences obtained codes for six (residues 9, 24, 31, 32, 43, 52) out of eleven residues forming P1, five (residues 11, 62, 65, 66, 69) out of seven forming P6 and five (residues 68, 69, 72, 73, 78) out of ten forming P9 [66].

In the alleles isolated, the non-synonymous substitutions in the PBR residues produced different variants of P1, P6 and P9, respectively; four variants were detected for P1 and P6, respectively, and two for P9 (Fig. 3). One variant of P1 (YHKFWT) and of P6 (NDTAN) were not detected in our previous study that covered a wide range of the Eurasian distribution of *L. europaeus* [45].

Each allele bore a combination of P1, P6 and P9 variants. A total of five combinations were detected in our samples. Surprisingly, alleles Leeu-DQA*14 and *53* that were detected in infected, yet alive individuals, and DQA*30 that was present in most infected and dead individuals, shared the same combination (YHEFWR/NETAN/YNILR). This particular combination was found to be the dominant combination in the north European populations and totally absent from the Anatolian ones [45]. Allele Leeu-DQA*54 bore that variant combination YHQFWA/NNTAG/YGIMR, which is present in high frequency in Greek populations and absent from the Anatolian ones. Allele Leeu-DQA*52 that appeared to support resistance to infection and increase survival, bore the newly identified P1 variant YHKFWT.

Antigenic peptide is anchored in the PBR through hydrogen bonds between PBR and antigen residues. Four such PBR residues (62, 68, 69, 76) are coded by exon 2, two of which are variable (62 and 69). Moreover, two out of 10 residues between positions 39–68 that are involved in TCR contact were variable (Fig. 3). Finally, five out of 10 residues responsible for αβ chain pairing were polymorphic (Fig. 3). So far, besides the variant combination, alleles Leeu-DQA*14, 30 and *53* shared the same TCR contact-, αβ chain pairing- and hydrogen bond-forming residues.

## Discussion

In this study, we examined the level of genetic diversity of the brown hare from different regions of Denmark with regard to the second exon of the MHC DQA locus, which is one of the most polymorphic class II loci. In addition, we aimed to perform an integrated analysis of the correlation of this genetic diversity with the susceptibility and/or resistance to EBHS. Although viral antigens are expected to be processed through the classical MHCI pathway, cumulative molecular data show that alternative pathways within antigen-presenting cells involving MHCII molecules function to increase retention and presentation of an as wide as possible variety of antigenic molecules (reviewed by Eisenlohr 2013 [70]). Moreover, Because of the acute pathogenesis described for EBHSV, it is likely that generation of humoral and other immune responses characteristic of infection with cytopathic viruses would dominate in EBHS, and that exogenous processing of viral peptides to MHC Class II would be important.

The overall variations for nucleotide differences for the second exon of the MHC DQA locus were clearly lower in Denmark than these assessed in other European countries (2.9% vs. 6.8%) but similar for amino acid differences (14.9% vs. 12.9%) [45]. Andersen et al. [71], analysing mitochondrial D-loop sequences of 369 brown hares from Denmark, found similar levels of genetic diversity between Danish and other European brown hare populations. Populations of the brown hare in Denmark as well as in Central Europe have been influenced by glaciation over the past 18 000 years. It is probable that these populations originated from a range of expansion events from southern refuges or refuges north east of the Black Sea that followed the retreat of the ice sheets at the end of the Pleistocene epoch, and their genetic structure was shaped during multiple genetic drift phenomena and anthropogenic effects (eg translocation and escapes from hare-farms) [71], [72], [73]. As a consequence of the founder effect, brown hare populations that originated from Pleistocene refugia have experienced a reduction in genetic diversity, since Central and Northern European populations exhibit lower levels of genetic diversity than populations from the Balkans or Anatolia, as revealed by the analysis of mtDNA [73] and allozymes [74].

Interestingly, the diversity of the DQA locus determined in this study was at variance with that proposed by the phylogeographic scenario above, because although Central and Northern European populations showed a similar or even higher diversity than some of the Balkan and Anatolian populations [45], this was not observed in hares from Denmark. Unexpectedly, the low DQA variation in Denmark did not fit into this expected frequency. Both Denmark and Great Britain are on the edge of the distributional range of the brown hare. However, in the latter, although the level of mtDNA diversity is extremely low in the brown hare [73], the brown hare population exhibited a substantial level of DQA polymorphism (e.g. eight alleles in 30 individuals; [45]), compared with the former (seven alleles in 158 individuals). On the other hand, as shown within the general European pattern where 54% of the alleles were population specific, unique alleles were also identified in 57% of the Denmark hares. Several factors, including occurrence of infectious agents and specific features of the mating system, might drive the evolution of MHC genes [46]. Nevertheless, it is probable that only the effects of pathogens vary both over time and among populations.

The influence of host MHC genotype on susceptibility and/or resistance to diseases has been established in multiple taxa [34], [39], [41], [75]–[80].Individuals from wild populations are often exposed to a diverse range of pathogens that vary both genetically and antigenically. It is the polymorphism at MHC loci that determines the diversity of foreign antigens recognised by the host immune system which in turn leads to specific immune responses. Studies in humans and infection of experimental animals provide support for heterozygote advantage [81]–[85]. However, there are reports which show no evidence for heterozygote advantage, but rather show disease resistance that is attributable to single alleles and therefore more consistent with frequency-dependent selection [85]–[90]. As in almost all the examined populations of brown hares from Europe and Anatolia [45], the observed heterozygosity in Denmark was also lower than that expected. Although, this study was based on animals from a population with a strong proportion of (apparent) homozygotes, it was very interesting that the proportion of heterozygous individuals within the group of EBHSV positive but apparently healthy hares was significantly higher than within the group of EBHSV positive but dead hares. However, the presence of small number of heterozygous individuals within the EBHSV positive group limited our ability to disentangle the effects of pure heterozygote advantage for resistance to this infection, because there is the possibility that this difference could be also attributed to genetic drift.

The selective advantage of rare alleles has been also proposed to be an important determinant of MHC variability in binding specific alleles [91]. Natural populations contain alleles in low frequency and several hypotheses have proposed that ‘balancing selection’ maintains such rare alleles in populations [92]. In the present study, four out of seven alleles (57.14%) were rare, with frequencies less than 0.05, and two of them were found solely in heterozygous state, as expected in the context of balancing selection [93]. Nevertheless, the hypothesis which postulates that polymorphism in the MHC might be maintained by a selective advantage of heterozygotes, is not supported by all population surveys of brown hares [45]. In a recent study of populations from Belgium and Austria, DQA heterozygosity was associated with other biological traits such as reproductive ability, where there was a clear tendency of heterozygotes, to produce a larger number of offspring [94]. Alternatively, the hypothesis of negative frequency-dependent selection could explain the maintenance of the observed high number of rare alleles. This hypothesis postulates that individuals carrying rare alleles have a potential advantage, which enable them to better resist new pathogen threats and therefore keeps those rare alleles in the gene pool instead of purging them.

Several studies suggest that specific MHC-II genotypes may confer resistance to a variety of pathogens in wild vertebrates and most of these studies found associations between certain MHC alleles and parasitism with single viral, bacterial or parasitic agents [95]–[98]. The association between specific alleles and parasite species is often taken as a support for the frequency-dependence hypothesis, particularly when rare MHC alleles are associated with increased resistance and common alleles with greater susceptibility [43], [95], [99]. In some cases, there is evidence that heterozygotes actually have reduced fitness relative to homozygotes [100], [101].

The most intriguing finding of this study was the potential association between the susceptibility (defined as the occurrence of deaths) to the virus infection and the most abundant allele Leeu-DQA*30. Also, resistance was possibly associated with the second most common allele Leeu-DQA*14. Even with the size limitations of the current data set, the high statistical significance of the findings pointed towards an important role for the alleles Leeu-DQA*30 and Leeu-DQA*14 in virus susceptibility and resistance, respectively. It is interesting to note that the “susceptibility” allele Leeu-DQA*30 was completely absent from populations of Anatolia (Turkey and Israel) and present in very low frequencies in few populations of Greece, whereas the same allele was the most common in almost all central and northern European populations [45]. This information could be of crucial importance for the co-evolution between EBHSV and hares MHC genes, considering that “central European” and “Greek” strains of the virus were found to be phylogenetically divergent [23], which was reconfirmed in our current study (average genetic distance 0.082 Danish vs other European and 0.075 Danish vs Greek).We hope that an ongoing study on the prevalence of infectious diseases, especially of EBHSV, among brown hares in Greece in association with the distribution of class II MHC alleles will shed more light on this issue.

Regarding the “resistance” allele Leeu-DQA*14; although absent from Anatolia, it was present in high frequencies throughout Europe [45], including Greece and Denmark. However, the hypothesis of negative frequency-dependent selection that could explain the maintenance of rare *DQA* alleles in Denmark population fails to explain the mechanism promoting the high prevalence of the “resistance” allele (the second most common in our study). In accord with the observations of others [102], the sampling time point may have had affected the data based on the cycling pattern of frequencies of both alleles and parasites due to host-parasite co-evolution. According to this, the sampling period could be a crucial parameter in the analysis of the assessed alleles’ frequencies, given that an advantageous and formerly rare allele could spread and reach high frequencies until the parasite evolves to avoid it [103]. The occurrence of the allele Leeu-DQA*14 in high frequencies in almost all European populations simultaneously favors this explanation.

Surprisingly, alleles Leeu-DQA*14 and Leeu-DQA*53 that were correlated with high survival of EBHSV infected animals shared the same amino acid residues contributing to PBR, TCR contact, hydrogen bonding and αβ pairing functions with Leeu-DQA*30, which was linked with high infection and low survival rates (Fig. 4, Table 1). This finding indicates that the potential contribution of these alleles to differential susceptibility to EBHSV should be sought outside α1 domain encoded by exon 2. DQα chains pair with DQβ ones to form the functional MHC molecule. Residues involved in the homodimerization stretch over the entire surface of the molecule and for α chains are located within α2 domain [66]. Thus, it is highly probable that Leeu-DQA*14, Leeu-DQA*53 and Leeu-DQA*30 possess different α2 domains that differentiate the palette of DQβ chains for homodimer formation resulting in MHC molecules with different antigen-presenting properties. In addition, antigen-presenting properties of DQ molecules appear to be less sharply defined compared with DR and DP, widening their binding repertoire. A study on the binding specificities of the six most common HLA-DQ molecules present world-wide revealed that some DQ anchor preferences represent a wide range of chemical specificities and indicated that peptide-pocket interactions might be less crucial for DQ binding and that the major determinant is that certain residues are forbidden, rather than a direct preference [104]. Furthermore, many residues of α2 domain impact contact with CD4 co-receptor, two of which are variant [66]. The possible structural differentiation between DQ molecules may reflect important immunoregulatory actions as certain regions of DQ molecules have been shown to exert immunosuppresory action by inhibiting activation or specific functions of CD4^+^ T-cells [105].

Deciphering the underlying mechanisms of brown hares’ susceptibility and/or resistance to the EBHS and identifying potential “susceptible” and/or “resistant” alleles has implications for management of hare populations in Europe. It is well established that along with other conservation management practices, restocking programs with animals bred in captivity have been widely used to stabilize or increase, for hunting purposes, brown hare populations in several European countries. As EBHS is a highly contagious and fatal disease that affects both wild and farmed hares, these findings must be seriously taken in to consideration by conservation authorities. Implications of these actions may include: a) wildlife management for hunting often causes a loss of genetic variation of game species and may lead to short-term reduction of fitness components [106], [107] and disease susceptibility [30], [108]; b) common and population-specific MHC alleles for both *DQA* [45] and *DRB* genes occurred in all so far studied European populations; c) EBHSV strains in Europe probably occurred in more than one phylogenetic lineages [23] and non indigenous strains could be easily imported to a country together with released imported hares and d) a potential association between susceptibility to the virus infection and specific *DQA* alleles, occurring with very different frequencies throughout Europe (from zero to very common) [45]. Therefore, there are multidimensional aspects to wildlife management and it should be strongly advised to reinforce and implement microbiological and genetic controls during restocking operations before any release or reintroduction of captive-bred animals.
